# Molecular and Physiological Effects of Magnesium–Polyphenolic Compound as Biostimulant in Drought Stress Mitigation in Tomato

**DOI:** 10.3390/plants11050586

**Published:** 2022-02-22

**Authors:** Haytham Hamedeh, Shaula Antoni, Lorenzo Cocciaglia, Valentina Ciccolini

**Affiliations:** Department of Research and Development, FertiGlobal Division, SCL Italia S.p.A, Via Fabio Filzi, 25/A, 20124 Milan, Italy; hamedeh@fertiglobal.com (H.H.); antoni@fertiglobal.com (S.A.); cocciaglia@fertiglobal.com (L.C.)

**Keywords:** ALPAN^®^, EnNuVi^®^, polyphenols, water deficit, abiotic stress alleviation

## Abstract

Plant biostimulants are being recognized as innovative tools to improve sustainable agricultural practices to mitigate the drastic effects of climate change, which is leading to a severe reduction in agricultural yields. In this work, a new biostimulant (EnNuVi^®^ ALPAN^®^) was evaluated for its effectiveness on tomato (*Solanum lycopersicum* Mill. cv. Rio Grande) plants subjected to water deficit conditions. The molecular effects were elucidated through transcriptomic RNA-seq and gene expression qPCR analysis and the physiological responses were evaluated through qualitative analysis of pigments and proline content, membrane stability, and lipid peroxidation. ALPAN^®^ was shown to adjust the transcriptional response by upregulating genes involved in source to sink carbohydrate metabolism and translocation, stomatal closure, and cell homeostasis. ALPAN^®^ was shown to mitigate the deteriorating effects of water deficit on the physiological status of the plants by stabilizing the levels of the photosynthetic pigments, regulating the accumulation of osmo-protectants, and preserving the cell wall lipid bilayer from oxidation. In conclusion, transcriptomic and physiological analysis provided insightful information on the biostimulant effects, indicating a positive role of ALPAN^®^ foliar application in alleviating the negative costs of water deficit.

## 1. Introduction

With the global population on the rise, it is expected to reach almost 10 billion by 2050 according to the United Nations [1], agricultural production is challenged to produce more while simultaneously facing a climate change disaster that is leading to the increase in extreme climatic events (drought, flooding, heat, salinity, etc.) either in frequency or intensity [2].

Drought, among these climatic events, is considered a major problem in crop production, and according to FAO (Food and Agriculture Organization), drought is responsible for almost 34% of total yield losses [3]. In addition to its effect on plant’s productivity, the decrease in water availability triggers a wide array of responses that range from molecular, physiological, and biochemical to ecological events [4,5]. Upon exposure to drought, plants become more vulnerable to cell injury due to the increase in cellular production of reactive oxygen species (ROS) that takes place in the mitochondria and chloroplast [6]. This spike in ROS functions as an alarming signal to trigger acclimation to the uprising stress [7,8]. Moreover, drought can damage various biochemical and physiological processes such as photosynthesis, respiration, ion uptake [9], carbohydrate metabolism [10,11], and protein post translation modifications [12]. In severe drought conditions, those damages become irreversible because of the disturbed metabolism and impaired photosynthetic apparatus and, hence, the eventual death of the plant occurs.

Due to their sessile nature, plants have evolved a wide range of mechanisms to respond to changes in their surrounding environment. Among such responses is the accumulation of plant’s secondary metabolites [13]. Polyphenols, which are the largest group of plant-specialized metabolites, are recognized as compounds involved in stress protection in plants [14]. Phenolic compounds, being a part of the phenylpropanoid pathway, are known to be produced in higher amounts under stress conditions as they play a significant role in controlling plant’s growth and development [15]. Additionally, nutrients fertilization has been proven to support plant growth and development under abiotic stress conditions by their direct role in the growth, metabolism, and biosynthesis of molecules in the plant cells that have a positive impact on a plant’s growth and development [16,17].

Among the available tools for farmers, plant-based biostimulants have gained more attention recently as low input crop management tools for sustainable agriculture. This type of biostimulants is being used to mitigate the negative and deleterious effects of water deficit under field/greenhouse conditions [18,19,20]. EnNuVi^®^ (acronym: Enhance, Nurture and Vitalize) is a recently patented plant-derived biostimulants technology based on the complexation of the plant-derived pool of polyphenols with essential nutritive element(s) (e.g., Cu, Mg, Mn, Zn).

The aim of this work was to characterize the response of tomato seedlings sprayed with ALPAN^®^, a magnesium-based EnNuVi^®^ product, by investigating the physiological and molecular changes induced by water deficit and the potential positive effects of the foliar spraying in the mitigation of the harmful consequences of water deficit conditions. The molecular mechanism was revealed through transcriptomic (RNA-seq) and gene expression analysis (qPCR), while the physiological response was evaluated through the quantification of proteins and metabolites related to photosynthetic activity, osmoregulation, and cell integrity.

## 2. Results

### 2.1. ALPAN^®^ Treatment Preserved Photosynthesis in Tomato Seedlings under Water Deficit Condition

The quantification of photosynthetic pigments involved in light absorption is a clue to the actual functional capacity of photosystems and, therefore, of the photosynthetic apparatus in general. Chlorophyll *a* (chl *a*) and chlorophyll *b* (chl *b*) can both bind to the light harvesting complex in the chloroplast to absorb blue and red light used for photosynthesis. Carotenoids, in turn, absorb blue light and are additionally involved in energy dissipation by heat through the xanthophyll cycle, quenching of ^1^O_2_ formed during photo-oxidation, and regulation of thylakoid membrane fluidity [21]. Chloroplast pigment content is one of the most frequently used indicators of the plant tolerance status when exposed to several abiotic conditions including drought [22].

**Figure 1:**
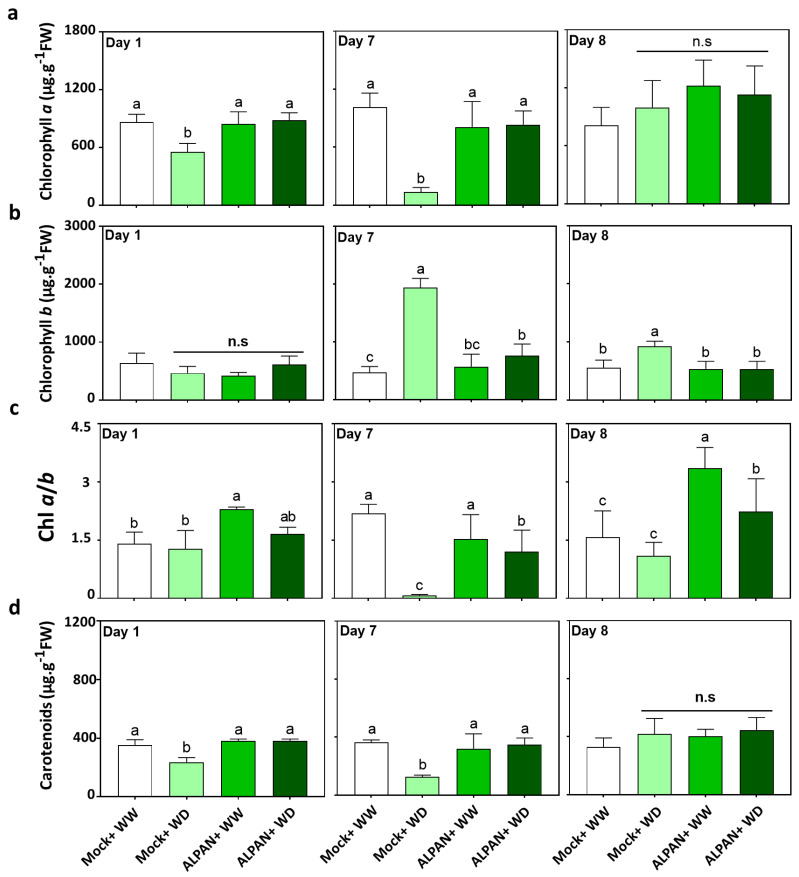
Concentrations of (**a**) Chlorophyll a, (**b**) Chlorophyll b, (**c**) chl *a*/*b* ratio and (**d**) carotenoids after 1, 7, and 8 days of the initiation of the water deficit stress. WW = well watered; WD = water deficit; FW = fresh weight; n.s = not significant. Error bars above histogram columns represent the standard deviation of four independent biological replicates in each experimental group at each sampling timepoint. The statistical data were analyzed with the use of the one-way ANOVA and Tukey’s post hoc tests, and a statistically significant difference (*p*-value < 0.05) is denoted by a different letter above a histogram column.

The mock water deficit (Mock + WD) plants showed a decrease in the level of chl *a* one day after the start of the water deficit, and this decrease was sharper when the water deficit duration was extended to seven days (Figure 1a). Nonetheless, one day after the start of recovery (day 8), the concentration of chl *a* returned to a level comparable to that of the no stressed plants (Figure 1a), while chl *b* showed a sharp accumulation in the Mock + WD plants (Figure 1b) as an attempt to compensate for the loss of chl *a* to maintain the minimal electron flow into the electron transfer chain. The reduction in chl *a* and the accumulation of chl *b* after seven days of stress, resulted in a very low chl *a*/*b* (Figure 1c).

Moreover, the decrease in chl *a* was accompanied by a gradual and significant decrease in carotenoids (Figure 1d), thus indicating a dysfunctional photosystem under water deficit conditions, which was restored to normality only when water was available again (day 8) (Figure 1d).

On the other hand, when ALPAN^®^ was applied, the plants showed greater ability to alleviate the negative effects of water deficit compared to those observed in the Mock-WD plants (Figure 1). ALPAN^®^-treated plants showed a better efficiency in the stabilization of the photosynthetic pigments (chlorophyll *a*, chlorophyll *b* and carotenoids) at all the timepoints (Figure 1). The results observed confirm that the application of ALPAN^®^ can contribute to improving the efficiency of photosynthesis by increasing or stabilizing the content of chloroplast pigments such as chl *a*, chl *b*, and carotenoids.

### 2.2. ALPAN^®^ Treatment Alleviated Cell Damage and Protected Cellular Membranes in Tomato Seedlings under Water Deficit Condition

The electrolyte leakage (EL) from plant tissues is often used as a parameter to assess cell integrity and for the initial detection of plant stress. Since membrane damage always results in an increased leakage of cytosolic constituents to the apoplastic space, higher values of EL implies lower membrane stability.

**Figure 2:**
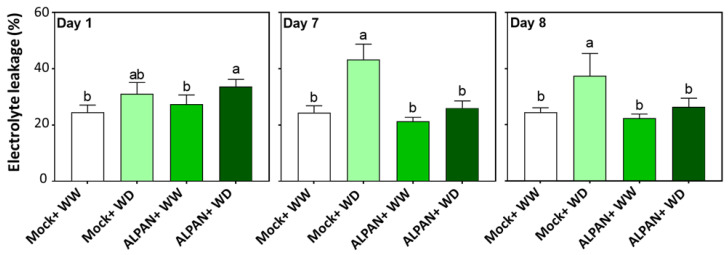
Electrolyte leakage determined after 1, 7, and 8 days of the initiation of the water deficit stress. WW = well watered, WD = water deficit. Error bars above histogram columns represent the standard deviation of four independent biological replicates in each experimental group at each sampling timepoint. The statistical data were analyzed with the use of the one-way ANOVA and Tukey’s post hoc tests, and a statistically significant difference (*p*-value < 0.05) is denoted by a different letter above a histogram column.

As noticed in Figure 2, Mock-WD plants showed a slight increase in the percentage of EL after one day of water deficit, whereas this increase was significantly higher after seven days of water deficit, and it remained high when regular watering was restored (day 8).

Plants exposed to water deficit and treated with ALPAN^®^ showed a significant increase in EL during the early periods of water deficit (Figure 2, day 1, dark green bars), whereas at days seven and eight, ALPAN^®^-treated plants showed a more significant reduction in EL, hence maintaining more stable cellular membrane.

To evaluate more directly the extent of cellular damage experienced by tomato plants under water deficit, the level of lipid peroxidation was estimated through the accumulation of malondialdehyde (MDA) as an end product generated by oxidation of polyunsaturated fatty acids. The oxidative degradation or lipid peroxidation is a chain reaction created by reactive oxygen species that influence lipids containing a carbon–carbon double bond resulting in cellular damage.

**Figure 3:**
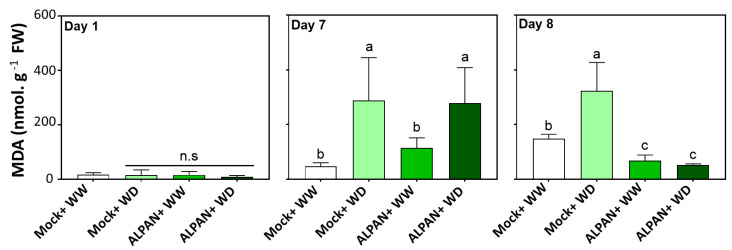
Malondialdehyde (MDA) concentration after 1, 7, and 8 days of the initiation of the water deficit stress. WW = well watered, WD = water deficit; FW = fresh weight; n.s = not significant. Error bars above histogram columns represent the standard deviation of four independent biological replicates in each experimental group at each sampling timepoint. The statistical data were analyzed with the use of the one-way ANOVA and Tukey’s post hoc tests, and a statistically significant difference (*p*-value < 0.05) is denoted by a different letter above a histogram column.

MDA concentrations were similar after one day of water deficit in all the experimental groups (Figure 3), whereas on day seven of water deficit, the Mock + WD plants showed an increase in MDA levels, similar to ALPAN + WD plants. This increase might indicate the occurrence of lipid peroxidation and membrane damage once the plant is exposed to a prolonged duration of water deficit. While the Mock+ WD plants could not revert this process when the regular watering was restored (day 8), ALPAN^®^-treated plants showed a significant reduction in MDA values. These results indicated that ALPAN^®^ application was efficient in avoiding irreversible damages in the cellular membrane.

### 2.3. ALPAN^®^ Treatment Modulated Proline Levels in Tomato Seedlings under Water Deficit Condition

Proline accumulation is one of the most important adaptative mechanisms for plants to cope with drought stresses, whereas higher levels can lead to adverse effects in maintaining the long-term tolerance mechanism.

**Figure 4:**
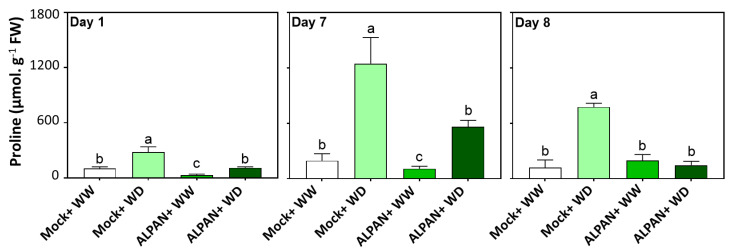
Proline concentration after 1, 7, and 8 days of the initiation of the water deficit stress. WW = well watered, WD = water deficit; FW = fresh weight. Error bars above histogram columns represent the standard deviation of four independent biological replicates in each experimental group at each sampling timepoint. The statistical data were analyzed with the use of the one-way ANOVA and Tukey’s post hoc tests, and a statistically significant difference (*p*-value < 0.05) is denoted by a different letter above a histogram column.

The Mock-WD plants accumulated higher proline levels after 1 day of stress, and this accumulation continued to increase to reach triple the levels on day seven; the proline level did not further increase after rewatering (day 8) of plants but remained high (Figure 4, light green bars).

By applying ALPAN^®^, proline accumulated less in the stressed plants at all experimental timepoints, and the restoration of the normal watering regime allowed the restoration of normal proline levels (Figure 4, dark green bars). Conversely, the Mock-WD plants continued to accumulate higher levels of proline even after 24 h of rewatering.

### 2.4. ALPAN^®^ Treatment Altered the Transcriptome in Tomato Seedlings under Water Deficit Condition

**Figure 5:**
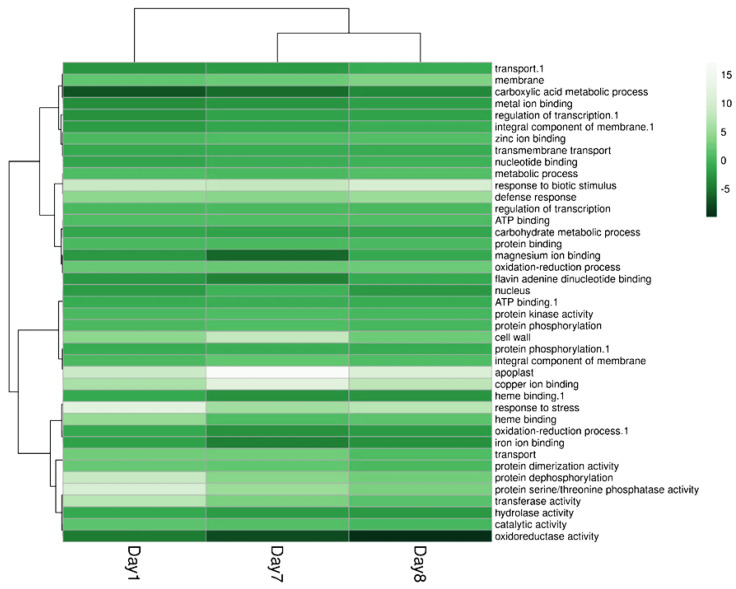
Hierarchical clustering of the main biological and molecular processes affected by ALPAN^®^ treatment under water deficit conditions (ALPAN + WD) in comparison to Mock + WD at day 1, 7, and 8. Heatmap was generated using Clustvis online tool.

RNA-seq data analysis revealed the molecular mechanism and the positive role of ALPAN^®^ in mitigating the adverse effects of drought stress. As indicated in Figure 5, plants treated with ALPAN^®^ were shown to orchestrate various biological processes and molecular functions involved in post translation modifications of proteins by modifying proteolysis and protein dephosphorylation. Serine/threonine phosphatase was upregulated during the stress period, in addition to modifications inflicted on the structure of cellular organelles to improve the apoplastic movement of water and carbohydrates.

**Figure 6:**
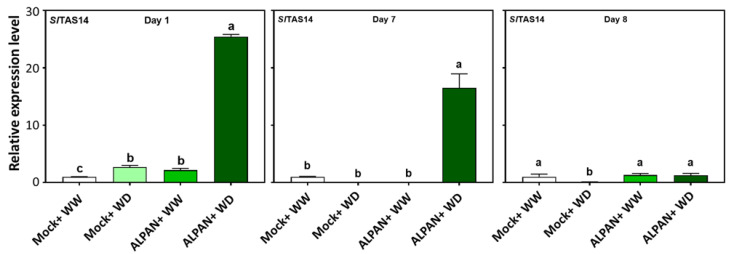
Expression levels of *SlTAS14* after 1, 7, and 8 of the initiation of the water deficit stress. WW = well watered, WD = water deficit. Error bars above histogram columns represent the standard deviation of four independent biological replicates in each experimental group at each sampling timepoint. The statistical data were analyzed with the use of the one-way ANOVA and Tukey’s post hoc tests, and a statistically significant difference (*p*-value < 0.05) is denoted by a different letter above a histogram column.

To confirm the results obtained from the RNA-seq analysis concerning the differentially expressed genes, we performed a gene expression analysis (qPCR) on specific genes involved in different functions under drought stress conditions. We quantified the expression of *SlTAS14*, a drought responsive marker gene [23,24] that encodes for late embryogenesis abundance (LEA) protein involved in responses to abscisic acid and water deprivation. ALPAN^®^-treated plants (ALPAN + WD) showed an upregulation in the expression of *SlTAS14* during short and long water deficit periods (Figure 6). On the other hand, Mock + WD plants showed a slight increase in expression only one day after the initiation of water deficit stress. Nonetheless, this expression disappeared after seven days of stress.

**Figure 7:**
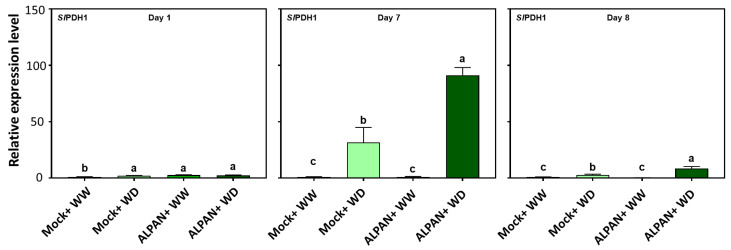
Expression levels of *SlPDH1* after 1, 7, and 8 days of the initiation of the water deficit stress. WW = well watered, WD = water deficit. Error bars represent the standard deviation of four independent biological replicates in each experimental group at each sampling timepoint. The statistical data were analyzed with the use of the one-way ANOVA and Tukey’s post hoc tests, and a statistically significant difference (*p*-value < 0.05) is denoted by a different letter above a histogram column.

Additionally, RNA-seq results were shown to modulate the expression of diverse metabolic processes involved in plant responses to water deficit. Among such processes is the tricarboxylic acid metabolism process (Krebs cycle) where the mitochondrial pyruvate dehydrogenase (*PDH*) complex is the convergence point that regulates the flux of pyruvate derived from glycolysis into the tricarboxylic acid [25]. Our results indicated that ALPAN^®^ was able to enhance the expression of the first subunit of the *SlPDH* complex during the later stages (day 7) of water stress (Figure 7).

**Figure 8:**
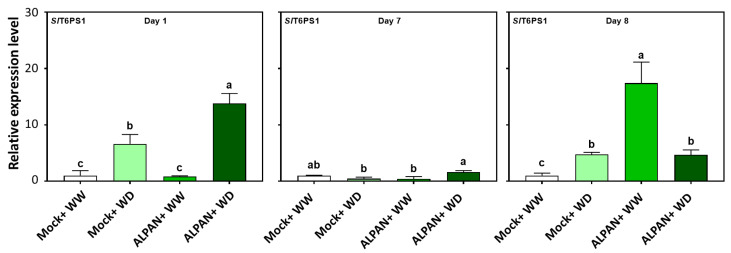
Expression levels of *SlT6PS1* after 1, 7, and 8 days of the initiation of the water deficit stress. WW = well watered, WD = water deficit. Error bars above histogram columns represent the standard deviation of four independent biological replicates in each experimental group at each sampling timepoint. The statistical data were analyzed with the use of the one-way ANOVA and Tukey’s post hoc tests, and a statistically significant difference (*p*-value < 0.05) is denoted by a different letter above a histogram column.

Another metabolic process identified through the RNA-seq analysis was the upregulation of glucose metabolism and trehalose biosynthesis. To confirm these results, we measured the expression of trehalose-6-phosphate-synthase 1 (*SlT6PS1*), the enzyme involved in trehalose-6-phosphate utilizing UDP-glucose and Glucose-6-phosphate as precursors [26]. Our results indicated a positive effect of ALPAN^®^ application in inducing the expression of *SlT6PS1* after one day of water deficit to increase the adaptation of young tomato seedlings to water deficit conditions (Figure 8, dark green bars).

**Figure 9:**
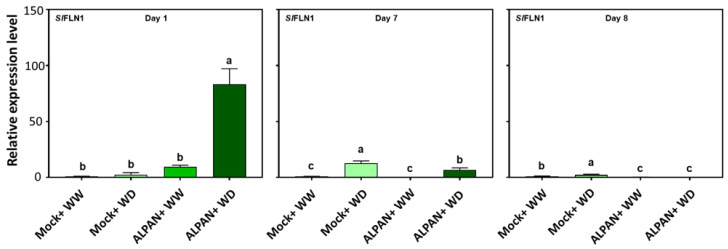
Expression levels of *SlFLN1* after 1, 7, and 8 days of the initiation of the water deficit stress. WW = well watered, WD = water deficit. Error bars above histogram columns represent the standard deviation of four independent biological replicates in each experimental group at each sampling timepoint. The statistical data were analyzed with the use of the one-way ANOVA and Tukey’s post hoc tests, and a statistically significant difference (*p*-value < 0.05) is denoted by a different letter above a histogram column.

ALPAN^®^ application was able to modify the chloroplastic redox regulatory mechanism. In *Arabidopsis thaliana* and *Nicotiana benthamiana* the interaction between plastidial thioredoxin z (*TRX*) and fructokinase-like 1 (*FLN1*) is essential for chloroplast development [27]. Our results showed an increase in the expression levels of *SlFLN1* (Figure 9, dark green bars) and *SlTRX* (data not shown) during the first day of drought stress.

**Figure 10:**
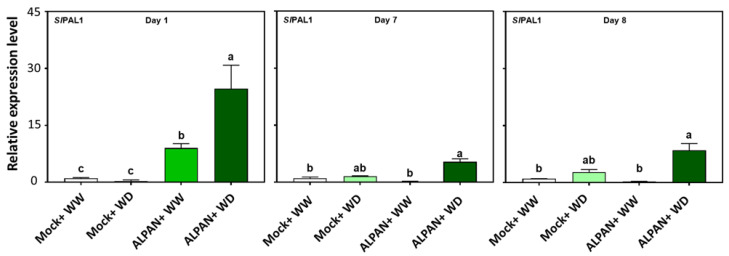
Expression levels of *SlPAL1* after 1, 7, and 8 days of the initiation of the stress. WW = well watered, WD = water deficit. Error bars above histogram columns represent the standard deviation of four independent biological replicates in each experimental group at each sampling timepoint. The statistical data were analyzed with the use of the one-way ANOVA and Tukey’s post hoc tests, and a statistically significant difference (*p*-value < 0.05) is denoted by a different letter above a histogram column.

ALPAN^®^ application positively affected the process of nitrogen assimilation, and our results indicated a positive effect on the expression of phenylalanine ammonia lyase (*SlPAL1*) (Figure 10). *SlPAL1* is an enzyme that helps in the catalyzation of L-phenylalanine to trans-cinnamic acid and ammonium, in which the conversion can be regarded as a critical step in inducing the metabolism in a plant [28].

## 3. Discussion

Plant biostimulants have gained deeper attention recently as sustainable tools to mitigate the negative effects of extreme weather events that result from climate change caused by human activities [29]. ALPAN^®^ is a natural biostimulant based on the complexation of plant-derived pool of polyphenols with an essential macronutrient, magnesium. Our study presents an insight into the positive effects of ALPAN^®^ treatment on tomato seedling exposed to mild water deficit conditions for seven days. ALPAN^®^ foliar application was shown to modulate the transcriptional and eco-physiological responses to short (one day) and longer (seven days) periods of water deficit conditions.

In the short term, ALPAN^®^ application was able to regulate processes involved in abscisic acid metabolism, non-structural carbohydrates metabolism, and source to sink translocation. Those three processes, among others, play a significant role in alleviating the negative effects of water shortage on plants [30,31]. In addition, ALPAN^®^ application showed a positive impact on stabilizing the levels of photosynthetic pigments that play a pivotal role in maintaining the process of photosynthesis under low water conditions and regulating a moderate increase in proline accumulation since an over-accumulation of proline can expose toxic effects on plant tissue [32].

In the long-term, our data indicate a crucial role for ALPAN^®^ in maintaining a functional photosynthetic apparatus through the stabilization of the levels of chlorophyll *a*, chlorophyll *b*, and total carotenoids after seven days of water deficit. Moreover, it was able to prevent ROS-mediated cell death that results from the loss of cell membrane integrity by keeping a low percentage of electrolyte leakage, an assay used to assess the stress-induced injury of plant tissues and an indicator of plant stress tolerance [33,34]. Additionally, RNA-seq showed that ALPAN^®^ was able to positively modify processes involved in: protein post translation modifications such as protein phosphorylation and dephosphorylation, cellular organelles modifications such as apoplast and nucleus, the sink to source translocation process, and nitrogen assimilation. Such processes are known to be negatively affected when plants are exposed to heterogenous environmental conditions such as drought.

In conclusion, ALPAN^®^ (based on EnNuVi^®^ technology) is an innovative, naturally derived biostimulant based on the combination of polyphenols and magnesium that can be added to the arsenal of tools aiming to serve farmers in mitigating the negative effects of reduced rainfall or the decreasing availability of irrigation water, which in turn affect plant growth and development leading to a great reduction in yield.

## 4. Materials and Methods

### 4.1. Plant Material, Growing Conditions, and Experimental Setup

Tomato (*Solanum lycopersicum*) plant (cv. Rio Grande) seeds were obtained from local seed supplier and germinated in the growth chamber (12 h light/12 h dark; 200 µm.m^−2^.S^−2^). One week after germination the plants were transplanted into 20 cm pots with a homogenous and equal weight of substrate mixture (two parts peat moss: one part perlite), and the watering was maintained until they reached 1 month old, a stage where the four leaves became well developed (BBCH = 104). Then, the plants (N = 32) were randomly assigned to four different experimental groups (four biological replicates; two plants per replicate): Mock well watered (Mock + WW), Mock water deficit (Mock + WD), ALPAN^®^ well watered (ALPAN^®^ + WW), and ALPAN^®^ water deficit (ALPAN + WD). Briefly, plants were sprayed with 20 mL of water (mock) or ALPAN^®^ (proprietary composition, SCL Italia SpA) at dilution rate of 0.1% *v/v*, one day prior to water deficit initiation (day 0; T1).

**Figure 11:**
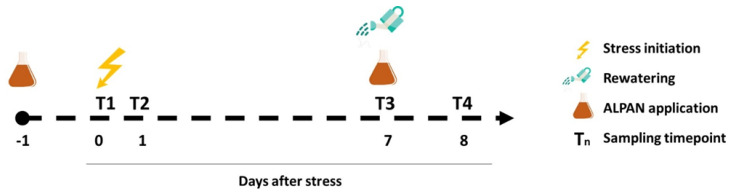
Schematic representation of the experimental design with the sampling time points for molecular and physiological analysis. Four biological replicates in each experimental group at each timepoint.

The first two samplings were sprayed one (T2) and seven (T3) days after the start of the water deficit, to assess the plant response after short and longer periods of water deficit conditions, respectively. Then at day 7, plants were re-watered to the full pot capacity and sprayed for a second treatment with water (mock) or ALPAN^®^. Then, one day after rewatering, a third sampling took place (day 8; T4) (Figure 11).

Leaf samples were collected and immediately frozen in liquid nitrogen and stored in −80 °C for further molecular and physiological analysis. Five independent experiments were carried out to perform each analysis (molecular analysis, pigments quantification, MDA quantification, EL estimation, proline quantification), applying the same experimental design with four independent biological replicates sampled from each experimental group at different sampling timepoint.

### 4.2. Chlorophyll and Carotenoids Quantification

Pigments were extracted according to Hiscox and Israelstam [35] with slight modifications. Briefly, 100 mg of frozen ground tissue were dissolved in 1 mL preheated DMSO using 1.5 mL Eppendorf tube and incubated for 15 min at 95 °C. A total of 900 µL of the supernatant was recovered into 2 mL Eppendorf tube through centrifugation (14,000 rpm for 10 min) and the pellet was redissolved with 1 mL preheated DMSO and incubated again for 15 min. Another 900 µL were recovered, then 200 µL of DMSO was added to obtain a final volume of 2 mL. The quantification of chlorophyll *a* and chlorophyll *b* were calculated according to Arnon [36], while carotenoids were calculated according to Kirk and Allen [37].

### 4.3. Membrane Integrity Assessment by Conductivity of Electrolyte Leakage

Membrane integrity was assessed by conductivity measurements of electrolyte leakage (EL) from four biological replicates, as reported in [38] with slight modifications. Briefly, 20 discs were cut from leaves punched using a cork borer and immersed in 10 mL double-deionized sterile water in 50 mL falcons. Samples were kept in agitation at room temperature with an orbital shaker (200 rpm) for 4 h, and conductivity was measured in the balanced bathing solution with a conductivity meter, this corresponds to the initial conductivity (C_i_). To determine the values corresponding to maximum electrolyte leakage from lysed leaf material, conductivity was then measured after samples were autoclaved and the liquid cooled down, this corresponds to the total conductivity (C_t_). Percent ion leakage was expressed as the ratio of the two values multiplied by 100.

### 4.4. Lipid Peroxidation by Quantification of Malondialdehyde Levels

Lipid peroxidation was analysed with the thiobarbituric acid test, which determines malondialdehyde (MDA) as end product of lipid peroxidation [39]. Briefly, 50 mg aerial tissue was homogenized in 1 mL frozen 80% ethanol (*v/v*) on ice. Following centrifugation at 16,000× g for 20 min at 4 °C, the supernatant (0.5 mL) was mixed with 0.5 mL 20% trichloroacetic acid (*w/v*) containing 0.65% thiobarbituric acid (*w/v*). The mixture was incubated at 95 °C for 30 min and then immediately cooled in an ice bath. After centrifugation at 10,000× *g* for 10 min at 4 °C, the absorbance of the supernatant was measured at 532 nm, subtracting the value for nonspecific absorption at 600 nm. The MDA concentration was calculated from the extinction coefficient 155 mM^−1^ cm^−1^.

### 4.5. Cellular Proline Quantification

Proline quantification was performed according to [40]. Briefly, 50 mg leaf tissue were ground and resuspended in 40% Ethanol and left overnight at 4 °C. The extract was then centrifuged and the 250 µL of the supernatant was mixed in 1.5 mL Eppendorf tube with 1000 µL of the reaction mix and 250 µL Ethanol (40%). The tube was well mixed, and the content was transferred to cuvette and quantification was performed using the peak absorbance wavelength (520 nm).

### 4.6. RNA Isolation, cDNA Library Construction, and Gene Expression Analysis

Total RNA was extracted from leaf samples using Qiagen RNA isolation kit (Qiagen, Germany) according to the manufacturer’s instructions. DNA was removed by digestion with RNase-free DNase, and RNA was purified and concentrated using an RNeasy column (Qiagen, Germany). RNA quality was evaluated by 1% agarose gel electrophoresis for 28 S/18 S rRNA band intensity (2:1) and Agilent 2100 Bioanalyzer. The samples were quantified using Nanodrop 2000 spectrophotometer (Thermo Fisher Scientific, Waltham, MA, USA).The A260/A280 nm ratios for all samples ranged between 1.8 and 2.1. Only the RNA samples with 260:280 ratio ranging between 1.9 and 2.1 and RNA integrity number (RIN)  >  8.0 were used for further analysis.

Equal amounts of RNA were subsequently subjected to genomic DNA removal by aid of the RQ1 DNase kit (Promega) and cDNA synthesis using the Maxima First Strand cDNA Synthesis Kit for RT-qPCR, with dsDNase (Product no. K1671, Life Technologies, Carlsbad, CA, USA), following the manufacturer’s instructions. Four biological replicates in each experimental group.

To quantify gene expression, qPCR analysis was carried out with the ABI Prism 7300 sequence detection system (Applied Biosystems, Waltham, MA, USA) using the Power Up SYBR Green master mix (Life Technologies). Relative quantification of the expression (qPCR analysis) of each individual gene was performed using the comparative threshold cycle method [41]. Elongation Factor 1 Alpha (*SlEF1α*) was used as internal control according to Lacerda et al. [42]. qRT-PCR analysis was performed on single samples collected at T2, T3, and T4. Table 1 shows the sequence of the forward and reverse primers used for each gene.

### 4.7. RNA-Seq Analysis and Library Construction

RNA-Seq libraries were prepared with Illumina TruSeq Stranded mRNA Sample Preparation Kit as per the manufacturer’s instructions. The experiment included two genotypes under two conditions (each data point pooled from 12 plants), which resulted in four RNA-Seq libraries. These libraries were sequenced on Illumina HiSeq 2000 platform (Illumina, San Diego, CA, USA) with 100 nucleotide pair-end reads.

### 4.8. Statistical Analysis

Data were statistically analyzed using GraphPad Prism version 8.0 software package and Tukey’s post hoc tests (*p* ≤ 0.05), using the treatments as a statistical parameter to determine significant differences among treatment means.

## 5. Patent

US patent n°: US11168035B2, Italian patent n°: 102016000126419.

## Figures and Tables

**Table 1:** The sequences for the primers used in the amplification of qPCR products.

Locus	Gene	Froward Primer	Reverse Primer	Product Size
Solyc01g108020	*SlEF1α*	TGGAATTGGAACTGTCCCCG	GAGCTTCGTGGTGCATCTCT	125
Solyc02g084850	*SlTAS14*	TCCCTACTCCCTGAACCTCC	CAGTCTTGCGCATTTGGTCT	144
Solyc09g011850	*SlFLN1*	AGTGCAAACAAGGGCTGTCA	TCGGAGCTAAGCAGTGAATCC	135
Solyc12g009400	*SlPDH1*	TGCTGCACGAGATCCTATCAG	TCATCCGCAAATTCGACAGC	132
Solyc10g086180	*SlPAL6*	TTTCCAGGGCACTCCCATTG	GTCGTTGACAAGCTCGGAGA	103
Solyc07g006500	*SlT6PS1*	GGTGTTTGCTCTGTTTATGGTGT	CAGAACAGTCATCAAGATTAAGCAG	146

## Data Availability

Available upon request to the authors.
